# Supplementary material for: Cis-Element Engineering Promotes the Expression of Bacillus subtilis Type I L-Asparaginase and Its Application in Food
Source: Int J Mol Sci. 2022 Jun 13;23(12):6588. doi: 10.3390/ijms23126588 (PMC9224341; doi:10.3390/ijms23126588)
Supplement: Supplementary file 1 [file ijms-23-06588-s001.zip › ijms-1755194-supplementary-updated.pdf]

Cis-Element Engineering Promotes the Expression of Bacillus subtilis Type I L-Asparaginase and Its Application in Food

Jiafeng Niu, Ruxue Yan, Juan Shen, Xiaoyu Zhu, Fanqiang Meng, Zhaoxin Lu, Fengxia Lu*

College of Food Science and Technology, Nanjing Agricultural University, Nanjing 210095, China

* Correspondence: lufengxia@njau.edu.cn; Tel.: 0086-25-84395963

Supplementary Data

Supplementary Table S1: Description and sources of vectors with different promoters used in this study.

Strains/Promoter	Sigma factor	Application	Source
P43	σ^A, σ^B	Recombinant vector	[12]
PaprE	σ^A	Recombinant vector	[52]
PyxiE	σ^A	Recombinant vector	[53]
PsecA	σ^A	Recombinant vector	[54]
Pylbp	σ^W	Recombinant vector	[55]
Phag	σ^D	Recombinant vector	[15]
PfusA	σ^A	Recombinant vector	[15]
PSacB	σ^B	Recombinant vector	[56]
PydzA	σ^D, σ^E, σ^F	Recombinant vector	[13]
PspoVG	σ^H	Recombinant vector	[57]
PsrfA	σ^H	Recombinant vector	[28]
PlytR	σ^A, σ^X	Recombinant vector	[57]
PftsH	σ^A	Recombinant vector	[57]
PyvyD	σ^B, σ^H	Recombinant vector	[57]
PsodA	σ^B	Recombinant vector	[13]
PamyE	σ^A	Recombinant vector	[52]
P43-P43	σ^A, σ^B-σ^A, σ^B	Recombinant vector	This work
PaprE-P43	σ^A-σ^A, σ^B	Recombinant vector	This work

PyvyD-P43	σ^B, σ^H-σ^A, σ^B	Recombinant vector	This work
PspoVG-P43	σ^H-σ^A, σ^B	Recombinant vector	This work
PaprE-PaprE	σ^A-σ^A	Recombinant vector	This work
P43-PaprE	σ^A, σ^B-σ^A	Recombinant vector	This work
PyvyD-PaprE	σ^B, σ^H-σ^A	Recombinant vector	This work
PspoVG-PaprE	σ^H-σ^A	Recombinant vector	This work
PyvyD-PyvyD	σ^B, σ^H-σ^B, σ^H	Recombinant vector	This work
P43-PyvyD	σ^A, σ^B-σ^B, σ^H	Recombinant vector	This work
PaprE-PyvyD	σ^A-σ^B, σ^H	Recombinant vector	This work
PspoVG-PyvyD	σ^H-σ^B, σ^H	Recombinant vector	This work
PSpoVG-PspoVG	σ^H-σ^H	Recombinant vector	This work
P43-PspoVG	σ^A, σ^B-σ^H	Recombinant vector	This work
PaprE-PspoVG	σ^A-σ^H	Recombinant vector	This work
PyvyD-PspoVG	σ^B, σ^H-σ^H	Recombinant vector	This work

Supplementary Table S2: Description and sources of different RBS and spacer sequences used in this study.

Strains	Promoter	RBS and spacer sequences	Source	Translation rate (au)
RBS 0	PaprE-PyvyD	GGGAGGCGTTCTTTG	PyvyD	4960.1
RBS 1	PaprE-PyvyD	AGGGGGATTTATT	PnprE	87846.79
RBS 2	PaprE-PyvyD	GTAAGAGAGGAATGTACAC	P43	8881.85
RBS 3	PaprE-PyvyD	AGGAGCGATTTACAT	PhpaII	11469.9
RBS 4	PaprE-PyvyD	AGGAGAGGGACGCGT	PaprE	20612.74
RBS 5	PaprE-PyvyD	AAAGGAGGAAGGATCA	pHT43(Pgrac)	85822.58
RBS 6	PaprE-PyvyD	AGGAGGTGTGTGTA	pMA0911	55958.16
RBS 7	PaprE-PyvyD	GGGAGGTATGACAAT	PsrfA	80498.66
RBS 8	PaprE-PyvyD	AGGTGGTGAACTACT	PspoVG	22194.66

RBS 9	PaprE-PyvyD	GGAGTGTCAAG	PamyE	11168.34
RBS 10	PaprE-PyvyD	AAGGAGATATACAT	pET30a	32138.63
RBS 11	PaprE-PyvyD	GGAGGTTATTTCA	PgroES	118319.57
RBS 12	PaprE-PyvyD	AGGAGATATACCATGGGCAGCAGC	pET28a	2985.39
RBS 13	PaprE-PyvyD	GTGAGGTGGATGCA	Pveg	85648.93
RBS 14	PaprE-PyvyD	GGAGAGAGGAA	PwapA	9090.42
RBS 15	PaprE-PyvyD	GGAGGCGTAGGC	RBS Calculator	82241.74
RBS 16	PaprE-PyvyD	GGAGGTAGGTAATAC	RBS Calculator	107421.95
RBS 17	PaprE-PyvyD	AGGAGGCACAACAT	RBS Calculator	139574.81
RBS 18	PaprE-PyvyD	TCAAGGAGGTTGGATT	RBS Calculator	187915.15
RBS 19	PaprE-PyvyD	GGAGGTTATACG	RBS Calculator	266472.30

RBS 20	PaprE-PyvyD	GGAGGTCATATTT	RBS Calculator	315092.76
RBS 21	PaprE-PyvyD	GGAGGTTTATTTT	RBS Calculator	360348.07
RBS 22	PaprE-PyvyD	GGAGGTTTTTTTA	RBS Calculator	409128.04

Supplementary Table S3: Oligonucleotides used in this study.

Primers	Sequence
PaprE-F	TTTTTTGAGCAACTGGATCCGTTCTTTTCTGTATGAAAATAGTTATTTCG
PaprE-R	AGAGCTACTTTCTTTTTCATTCTTTACCCTCTCCTTTTAAAAAAATTCAG
PyxiE-F	TTTTTTGAGCAACTGGATCCAATTGAAGCGCGCGAAGC
PyxiE-R	AGAGCTACTTTCTTTTTCATGCTCTTCCCGCCTTTCGG
PsecA-F	TTTTTTGAGCAACTGGATCCGGACATCGTCCGTCAGAAACG
PsecA-R	AGAGCTACTTTCTTTTTCATTTATAACGCTCCTCTATCATCACACG
Pylbp-F	TTTTTTGAGCAACTGGATCCGTCACAACAGTCACGTCGTGATAA
Pylbp-R	AGAGCTACTTTCTTTTTCATACAAATCTCCCCCTTTGTTGTTT
Phag-F	TTTTTTGAGCAACTGGATCCGGATTTTTTTATTTTTGTATTAACAAAATCAGAGAC
Phag-R	AGAGCTACTTTCTTTTTCATTGTTTTGTTCCTCCCTGAATATGTT
PfusA-F	TTTTTTGAGCAACTGGATCCTTGATTTTGCCGCTTAACTCAAG
PfusA-R	AGAGCTACTTTCTTTTTCATTCTAAAATCCTCCTTAAGAGCTTTTAAT
PSacB-F	TTTTTTGAGCAACTGGATCCGATATTTTCTGAATTGTGATTAAA

	AAGGC
PSacB-R	AGAGCTACTTTCTTTTTCATCGTTCATGTCTCCTTTTTTATGTACTG
PydzA-F	TTTTTTGAGCAACTGGATCCCCCGTTCTGTTACAGATGGAG
PydzA-R	AGAGCTACTTTCTTTTTCATAAATCGCCCTCCTTGTGGA
PspoVG-F	TTTTTTGAGCAACTGGATCCACGGACAATATTTTGACACTCACAA
PspoVG-R	AGAGCTACTTTCTTTTTCATAGTAGTTCACCACCTTTTCCCTATATAA
PsrfA-F	AAGACAAATTCTGCTACTAATGATAGGTGGTATGTTTTCGCTTG
PsrfA-R	TCTTTGTCGCCGCTGCTGCACATGTGTACATTCCTCTCTTACCTATAATG
PlytR-F	TTTTTTGAGCAACTGGATCCCTAACCCTACATAAGTACCTTCTTTTGTT
PlytR-R	AGAGCTACTTTCTTTTTCATCCTTTGCACCTCGTCTGTTAAAT
PftsH-F	TTTTTTGAGCAACTGGATCCAGATTAGGATTGCGAGCAGCA
PftsH-R	AGAGCTACTTTCTTTTTCATCATAACCCTCCAGCATGTTTAAAA
PyvyD-F	TTTTTTGAGCAACTGGATCCGATCAATTGGTCTCTTTCTCTTTTCC
PyvyD-R	AGAGCTACTTTCTTTTTCATCAAAGAACGCCTCCCTTTTATTA
PsodA-F	TTTTTTGAGCAACTGGATCCGAAATGCTGGCGGCAGGT
PsodA-R	AGAGCTACTTTCTTTTTCATGATAATTCCTCCTTAGTATATATGTA

	CTGAAATG
PamyE-F	TTTTTTGAGCAACTGGATCCTAGAGTGATTGTGATAATTTTAAATGTAAGCG
PamyE-R	AGAGCTACTTTCTTTTTCATTCTTGACACTCCTTATTTGATTTTTTG
V-F	ATGAAAAAGAAAGTAGCTCTTATTACAACA
V-R	GGATCCAGTTGCTCAAAAAAATC
P43-P43-F	TTTTTTGAGCAACTGGATCCTGATAGGTGGTATGTTTTCGCTTG
P43-P43-R	CGAAAACATACCACCTATCATTATATTTTACATAATCGCGCGC
PaprE-P43-F	TTTTTTGAGCAACTGGATCCGTTCTTTTCTGTATGAAAATAGTTATTTCG
PaprE-P43-R	CGAAAACATACCACCTATCACTTACTTAAAAGACTATTCTGTGAATTTATTGT
PyvyD-P43-F	TTTTTTGAGCAACTGGATCCGATCAATTGGTCTCTTTCTCTTTTCC
PyvyD-P43-R	CGAAAACATACCACCTATCAATTTCTCTTTTACCCTTTACAATTCCT
PspoVG-P43-F	TTTTTTGAGCAACTGGATCCTGCGGAAGTAAACGAAGTGTACG
PspoVG-P43-R	CGAAAACATACCACCTATCGTGTATCAATTCCACGATTTTTTCTG
PaprE-PaprE-F	TTTTTTGAGCAACTGGATCCGTTCTTTTCTGTATGAAAATAGTTATTTCG
PaprE-PaprE-R	ATTTTCATACAGAAAAGAACCTTACTTAAAAGACTATTCTGTGA

	ATTTATTGT
P43-PaprE-F	TTTTTTGAGCAACTGGATCCTGATAGGTGGTATGTTTTCGCTTG
P43-PaprE-R	ATTTTCATACAGAAAAGAACTTATATTTTACATAATCGCGCGC
PyvyD-PaprE-F	TTTTTTGAGCAACTGGATCCGATCAATTGGTCTCTTTCTCTTTTC
	C
PyvyD-PaprE-R	ATTTTCATACAGAAAAGAACATTTCTCTTTTACCCTTTACAATTC
	CT
PspoVG-PaprE-F	TTTTTTGAGCAACTGGATCCTGCGGAAGTAAACGAAGTGTACG
PspoVG-PaprE-R	ATTTTCATACAGAAAAGAACGTGTATCAATTCCACGATTTTTTCT
	G
PyvyD-PyvyD-F	TTTTTTGAGCAACTGGATCCGATCAATTGGTCTCTTTCTCTTTTC
	C
PyvyD-PyvyD-R	GAGAAAGAGACCAATTGATCATTTCTCTTTTACCCTTTACAATTC
	CT
P43-PyvyD-F	TTTTTTGAGCAACTGGATCCTGATAGGTGGTATGTTTTCGCTTG
P43-PyvyD-R	GAGAAAGAGACCAATTGATCTTATATTTTACATAATCGCGCGC
PaprE-PyvyD-F	TTTTTTGAGCAACTGGATCCGTTCTTTTCTGTATGAAAATAGTTA
	TTTCG
PaprE-PyvyD-R	GAGAAAGAGACCAATTGATCCTTACTTAAAAGACTATTCTGTGA
	ATTTATTGT
PspoVG-PyvyD-F	TTTTTTGAGCAACTGGATCCTGCGGAAGTAAACGAAGTGTACG
PspoVG-PyvyD-R	GAGAAAGAGACCAATTGATCGTGTATCAATTCCACGATTTTTTC

	TG
PSpoVG-PspoVG-F	TTTTTTGAGCAACTGGATCCTGCGGAAGTAAACGAAGTGTACG
PSpoVG-PspoVG-R	AGTGTCAAAATATTGTCCGTGTGTATCAATTCCACGATTTTTTCTG
P43-PspoVG-F	TTTTTTGAGCAACTGGATCCTGATAGGTGGTATGTTTTCGCTTG
P43-PspoVG-R	AGTGTCAAAATATTGTCCGTTTATATTTTACATAATCGCGCGC
PaprE-PspoVG-F	TTTTTTGAGCAACTGGATCCGTTCTTTTCTGTATGAAAATAGTTATTTCG
PaprE-PspoVG-R	AGTGTCAAAATATTGTCCGTCTTACTTAAAAGACTATTCTGTGAATTTATTGT
PyvyD-PspoVG-F	TTTTTTGAGCAACTGGATCCGATCAATTGGTCTCTTTCTCTTTTCC
PyvyD-PspoVG-R	AGTGTCAAAATATTGTCCGATTTCTCTTTTACCCTTTACAATTCCT
Mutant 1-F	AGCAGGAATTGTAAAGGGTATAAGAG
Mutant 1-R	CCCTTTACAATTCCTGCTTAAACATA
Mutant 2-F	TGTAAAGGGTAAAAGAGAATTAGATACATATC
Mutant 2-R	CTCTTTTACCCTTTACAATACCTG
Mutant 3-F	GCAGGTATTGTAAAGGGTATAAGAGAATTAGATACATATC
Mutant 3-R	CCCTTTACAATACCTGCTTAAACATAAATCTTT
RT-BlA-F	TTCAGGACGGCAGCTATGAC
RT-BlA-R	TATATCTGGGCTGCACGCTG

16 S-F	GACGAAAGTCTGACGGAGCA
16 S-R	TGCCACCTACGTATTACCGC
RBS 1-F	AAGGGGGATTTATTATGAAAAAGAAAGTAGCTCTTATTACAACA
RBS 1-R	CATAATAAATCCCCCTTTTTATTAAGGATATGTATCTAATTCTCTTATACC
RBS 2-F	GTAAGAGAGGAATGTACACATGAAAAAGAAAGTAGCTCTTATTACAACA
RBS 2-R	GTACATTCCTCTCTTACTTTTATTAAGGATATGTATCTAATTCTCTTATACC
RBS 3-F	AAGGAGCGATTTACATATGAAAAAGAAAGTAGCTCTTATTACAACA
RBS 3-R	TATGTAAATCGCTCCTTTTTATTAAGGATATGTATCTAATTCTCTTATACC
RBS 4-F	AAGGAGAGGGACGCGTATGAAAAAGAAAGTAGCTCTTATTACAACA
RBS 4-R	TACGCGTCCCTCTCCTTTTTATTAAGGATATGTATCTAATTCTCTTATACC
RBS 5-F	AAAAGGAGGAAGGATCAATGAAAAAGAAAGTAGCTCTTATTACAACA
RBS 5-R	TTGATCCTTCCTCCTTTTTTTATTAAGGATATGTATCTAATTCTCTTATACC
RBS 6-F	AAGGAGGTGTGTGTAATGAAAAAGAAAGTAGCTCTTATTACAA

	CA
RBS 6-R	CATTACACACACCTCCTTTTTATTAAGGATATGTATCTAATTCTCTTATACC
RBS 7-F	AGGGAGGTATGACAATATGAAAAAGAAAGTAGCTCTTATTACAACA
RBS 7-R	AGGGAGGTATGACAATATGAAAAAGAAAGTAGCTCTTATTACAACA
RBS 8-F	AGGGAGGTATGACAATATGAAAAAGAAAGTAGCTCTTATTACAACA
RBS 8-R	TAGTAGTTCACCACCTTTTTATTAAGGATATGTATCTAATTCTCTTATACC
RBS 9-F	AAAGGAGTGTCAAGATGAAAAAGAAAGTAGCTCTTATTACAACA
RBS 9-R	CATCTTGACACTCCTTTTATTAAGGATATGTATCTAATTCTCTTATACC
RBS 10-F	AAAGGAGATATACATATGAAAAAGAAAGTAGCTCTTATTACAACA
RBS 10-R	CATATGTATATCTCCTTTTTTATTAAGGATATGTATCTAATTCTCTTATACC
RBS 11-F	AGGAGGTTATTTCAATGAAAAAGAAAGTAGCTCTTATTACAACA
RBS 11-R	CATTGAAATAACCTCCTTTTATTAAGGATATGTATCTAATTCTCTTATACC

RBS 12-F	AGATATACCATGGGCAGCAGCATGAAAAAGAAAGTAGCTCTTATTACAACA
RBS 12-R	GCTGCCCATGGTATATCTCCTTTTTATTAAGGATATGTATCTAATTCTCTTATACC
RBS 13-F	AGTGAGGTGGATGCAATGAAAAAGAAAGTAGCTCTTATTACAACA
RBS 13-R	CATTGCATCCACCTCACTTTTATTAAGGATATGTATCTAATTCTCTTATACC
RBS 14-F	AAAGGAGAGAGGAAATGAAAAAGAAAGTAGCTCTTATTACAACA
RBS 14-R	CATTTCCTCTCTCCTTTTATTAAGGATATGTATCTAATTCTCTTATACC
RBS 15-F	AAAAGGAGGCGTAGGCATGAAAAAGAAAGTAGCTCTTATTACAACA
RBS 15-R	ATGCCTACGCCTCCTTTTATTAAGGATATGTATCTAATTCTCTTATACC
RBS 16-F	AGGAGGTAGGTAATACATGAAAAAGAAAGTAGCTCTTATTACAACA
RBS 16-R	TGTATTACCTACCTCCTTTTATTAAGGATATGTATCTAATTCTCTTATACC
RBS 17-F	AAGGAGGCACAACATATGAAAAAGAAAGTAGCTCTTATTACAACA

RBS 17-R	CATATGTTGTGCCTCCTTTTTATTAAGGATATGTATCTAATTCTCT TATACC
RBS 18-F	ATCAAGGAGGTTGGATTATGAAAAAGAAAGTAGCTCTTATTACA ACA
RBS 18-R	TAATCCAACCTCCTTGATTTTATTAAGGATATGTATCTAATTCTCT TATACC
RBS 19-F	AAAGGAGGTTATACGATGAAAAAGAAAGTAGCTCTTATTACAA CA
RBS 19-R	CATCGTATAACCTCCTTTTATTAAGGATATGTATCTAATTCTCTTAT ACC
RBS 20-F	AGGAGGTCATATTTATGAAAAAGAAAGTAGCTCTTATTACAACA
RBS 20-R	CATAAATATGACCTCCTTTTATTAAGGATATGTATCTAATTCTCTT ATACC
RBS 21-F	AGGAGGTTTATTTTATGAAAAAGAAAGTAGCTCTTATTACAACA
RBS 21-R	CATAAAATAAACCTCCTTTTATTAAGGATATGTATCTAATTCTCTT ATACC
RBS 22-F	AGGAGGTTTTTTTAATGAAAAAGAAAGTAGCTCTTATTACAACA
RBS 22-R	CATTAAAAAAACCTCCTTTTATTAAGGATATGTATCTAATTCTCTT ATACC

Supplementary Figure S1: Acrylamide levels in potato chips subjected to different treatments. (A) The control group; (B) The blanched group; (C) the BlAase treatment group; (D) the combined of blanching and BlAase treatment group.

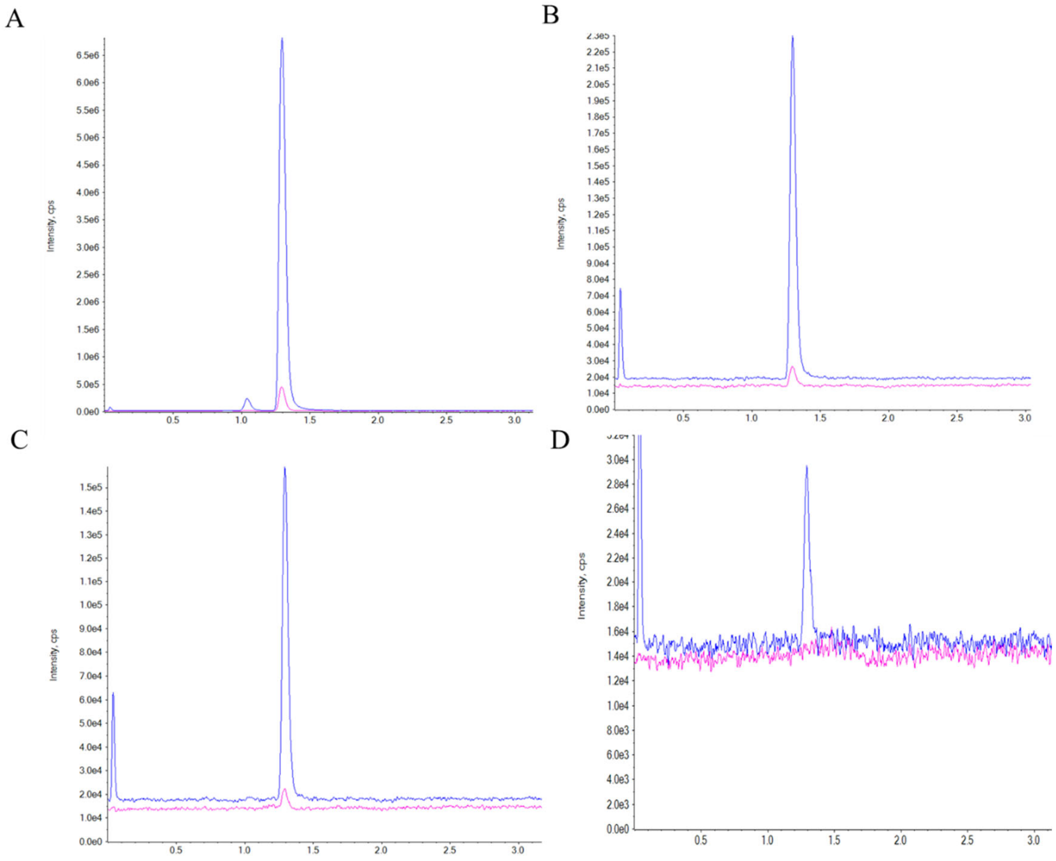

Figure S1
